# The major barriers to evidence‐informed conservation policy and possible solutions

**DOI:** 10.1111/conl.12564

**Published:** 2018-05-08

**Authors:** David C. Rose, William J. Sutherland, Tatsuya Amano, Juan P. González‐Varo, Rebecca J. Robertson, Benno I. Simmons, Hannah S. Wauchope, Eszter Kovacs, América Paz Durán, Alice B. M. Vadrot, Weiling Wu, Maria P. Dias, Martina M. I. Di Fonzo, Sarah Ivory, Lucia Norris, Matheus Henrique Nunes, Tobias Ochieng Nyumba, Noa Steiner, Juliet Vickery, Nibedita Mukherjee

**Affiliations:** ^1^ Department of Geography University of Cambridge Downing Place Cambridge CB2 3EN United Kingdom; ^2^ School of Environmental Sciences University of East Anglia Norwich Research Park Norwich NR4 7TJ United Kingdom; ^3^ Department of Zoology University of Cambridge The David Attenborough Building, Pembroke Street Cambridge CB2 3QZ United Kingdom; ^4^ Centre for the Study of Existential Risk University of Cambridge 16 Mill Lane Cambridge CB2 1SB; ^5^ Birdlife International The David Attenborough Building Pembroke Street Cambridge CB2 3QZ United Kingdom; ^6^ University of Cambridge Institute for Sustainability Leadership 1 Trumpington Street Cambridge CB2 1QA United Kingdom; ^7^ ARC Centre of Excellence for Environmental Decisions University of Queensland St Lucia 4072 Australia; ^8^ UN World Conservation Monitoring Centre 219 Huntingdon Road Cambridge CB3 0DL; ^9^ Forest Ecology and Conservation Group, Department of Plant Sciences University of Cambridge Downing Street Cambridge CB2 3EA United Kingdom; ^10^ Luc Hoffmann Institute c/o WWF International Avenue du Mont Blanc 1196 Gland Switzerland; ^11^ Centre for Science and Policy 10 Trumpington St. Cambridge CB2 1QA United Kingdom; ^12^ RSPB Centre of Conservation Science Royal Society for the Protection of Birds, The Lodge Sandy Bedfordshire SG19 2DL United Kingdom; ^13^ Centre for Ecology and Conservation, College of Life and Environmental Sciences University of Exeter Penryn Cornwall TR10 9FE United Kingdom; ^14^ Corvinus University of Budapest Fővám tér 8 Budapest 1093 Hungary

**Keywords:** conservation policy, evidence‐based conservation, evidence‐informed conservation, knowledge exchange, political science, science communication, science‐policy

## Abstract

Conservation policy decisions can suffer from a lack of evidence, hindering effective decision‐making. In nature conservation, studies investigating why policy is often not evidence‐informed have tended to focus on Western democracies, with relatively small samples. To understand global variation and challenges better, we established a global survey aimed at identifying top barriers and solutions to the use of conservation science in policy. This obtained the views of 758 people in policy, practice, and research positions from 68 countries across six languages. Here we show that, contrary to popular belief, there is agreement between groups about how to incorporate conservation science into policy, and there is thus room for optimism. Barriers related to the low priority of conservation were considered to be important, while mainstreaming conservation was proposed as a key solution. Therefore, priorities should focus on convincing the public of the importance of conservation as an issue, which will then influence policy‐makers to adopt pro‐environmental long‐term policies.

## 1 CHALLENGES FOR EVIDENCE‐INFORMED CONSERVATION POLICY

Loss of biodiversity is occurring at accelerated rates. Although there are uncertainties associated with the causes of biodiversity loss (Game, Meijaard, Sheil, & McDonald‐Madden, 2014), there is evidence that a range of conservation interventions are effective (Sutherland, Dicks, Ockendon, Smith, 2017). Many articles, however, highlight a gap between scientific evidence and policy, suggesting disagreement between the priorities of research scientists and decision‐makers (e.g., Arlettaz et al., 2010), with one study even accusing decision‐makers of “evidence complacency” (Sutherland & Wordley, 2017). Various processes are underway to improve the link between science and policy, including IPBES, and also the EU EKLIPSE mechanism, where selected scientists and practitioners resolve questions posed by policy‐makers. To enhance the likelihood of success of such science‐policy initiatives, research on the key barriers and solutions to the uptake of conservation science in policy is important.

Various publications note that scientific knowledge is just one factor in policy‐making (Marshall et al., 2017; Rose, Brotherton, Owens, & Pryke, 2016). In response, research has sought to increase the influence of science. These include techniques to link science and policy (e.g., Cvitanovic et al., 2015; Neßhöver et al., 2016), training scientists and policy‐makers to understand mutual workflows (Bainbridge, 2014), encouraging collaborative inter‐disciplinary research (Adams & Sandbrook, 2013; Young et al., 2014), and telling policy‐relevant stories (Cook, Mascia, Schwartz, Possingham, & Fuller, 2013; Rose, 2015; Sarkki et al., 2014). Solutions, though, have often been studied with little attention to their context dependencies (Kovacs & Pataki, 2016) (i.e. whether the same solutions will work everywhere especially if the problems are different), nor indeed has the majority of social science work at the science‐policy interfaces been solution‐oriented (Watts, 2017).

Furthermore, most studies on conservation science‐policy interfaces have been based on a relatively small number of respondents from Western democracies. Since gaps between science and policy may arise from cultural and/or social barriers (Amano, González‐Varo, & Sutherland, 2016), in addition to political and institutional factors (Owens, 2015), geographical bias can contribute to a misunderstanding of issues.

This research addresses the perceptions of different stakeholders about the relative importance of barriers to the consideration of evidence in decisions about conservation, placing the emphasis on identifying solutions to highly ranked barriers. Primary data was collected through multiple surveys in two phases across three groups of global respondents: people in policy positions, practitioners, and research scientists.^1^ The aims of the surveys were to understand the key barriers preventing the use of conservation science in policy, and to highlight potential solutions to overcome them.

## 2 SURVEY

The survey consisted of two phases (scoping survey followed by a global online survey translated into six languages). We briefly explain the stages involved in each of the two phases below. For more detailed information about methodology, including categorization, coding, survey dissemination, and sensitivity analyses, please see the supplementary material (S1 and Figure S1).

### 2.1 Phase 1: scoping

This survey (S2) had two iterations.

#### 2.1.1 Scoping survey 1

The first survey was distributed at a conference on conservation decision‐making. Respondents were asked to (1) select a role, (2) name three barriers preventing the use of conservation science in policy‐making, and (3) suggest solutions for the proposed barriers. The barriers and solutions sections were left open‐ended such that respondents were not constrained by our beliefs.

#### 2.1.2 Scoping survey 2

This was followed by a second survey that asked the same questions, but added questions relating to country of work, and their number of years of experience in a conservation role. This was distributed throughout other networks globally. In total, 134 responses were gained^2^ from 30 countries and open‐ended answers to both the barriers and solutions question were pooled and coded into categories (S3). The categories were ranked according to the number of times they were mentioned in both of the scoping surveys. This led to a top ten list for barriers and solutions. A list of the most highly ranked solutions was also developed (Table S3).

### 2.2 Phase 2: Online survey

A second online survey was created based on the answers provided in Phase 1 and translated into five other languages. In the second phase, the survey was mostly close‐ended (S4). The respondents were asked to score each of the top ten barriers and corresponding solutions from Phase 1 on a Likert scale of 1 (not important) to 8 (very important). The list of solutions for each barrier was based on the responses to the Phase 1 survey, but did not include every solution mentioned for each barrier (see S1). A range of approaches were used to disseminate the survey (e.g., known networks, social media, e‐mail lists).

## 3 MODELS

Cumulative link models were applied to test the relationship between the score of each barrier/solution (as ordinal response variables) and two explanatory variables: barrier/solution identity (see Table 1) and the role of respondents (policy position/practitioners/academics), as well as their interaction. The significance level of each term was derived from likelihood ratio tests and deviance for each term was also calculated, following Christensen (2015a). To rank the overall importance among distinct barriers and solutions, we calculated the mean of the median scores across the three roles for each barrier/solution. The aim of using the mean of medians, instead of the overall median per barrier/solutions was to control for the difference in the sample size across the different roles. We used the Kendall's rank correlation coefficient (*τ*) to test–in each of the three studied roles–for positive relationships between the percentage of respondents that experienced each barrier and the median barrier score. We thus performed one‐tailed tests because we expected these relationships to be positive. Sensitivity analyses were also performed to test whether scoring was affected by other covariates. The analysis was conducted in R (R Core Team, 2016) and cumulative link models were implemented with the R package ordinal (Christensen, 2015b).

**Table 1:** Top 10 barriers and selected solutions from phase one (not in quantitative order of phase one ranking here, see S3 for this)

Barrier number/name	Proposed solutions to each barrier
1. Lack of policy relevant science	Ask policy relevant questions from start of project, including policy‐makers Better incentives for academics to focus on policy/practice relevant research Embed young scientists in the field and train them on importance of real world science application Improve policy education of young scientists/scientists (e.g., through job shadowing, graduate training) More collaboration between scientists and policy‐makers (e.g., meetings, seminars, projects)
2. Conservation not a political priority	Demonstrate benefits of conservation (including economic value) Develop different measures of prosperity other than just GDP/economy Improve policy education of young scientists/scientists (e.g., through job shadowing, graduate training) More scientists working in/with media to engage policy‐makers and public Train policy‐makers in conservation science to help them see the importance of conservation
3. Mismatch of timescales	Better science advocacy from scientists Dedicated office at research institutions to help researchers communicate key information Encourage government departments to share reading of scientific outputs Encourage the strategic use of science for long‐term policy‐making Set up government advisory body that spans political timescales
4. Complex, uncertain problems	Better communication of uncertainty More transparency about uncertainty Standardize methods and indicators for conservation to improve communication Train scientists in a variety of communication skills Transdisciplinary research to be encouraged
5. Policy‐makers do not understand science	Better science education in schools and universities to improve science literacy of population More knowledge brokers (individuals to bridge the gap between science and policy) and system for it More scientists working in media to engage policy‐makers and public Tailor evidence to audience—e.g., blogs, summaries, simple language, open access, policy briefs, infographics Train policy‐makers in science
6. Lack of funding for conservation science	Better incentives for academics to focus on policy/practice relevant research Demonstrate benefits of conservation (including economic value) More collaboration between scientists and policy‐makers (e.g., meetings, seminars, projects) Permanent budget for environmental policy‐making
7. Priority of the private sector's agenda over conservation	Better science advocacy Demonstrate benefits of conservation (including economic value) Include industry and private sector in research Provide evidence‐based argument to counter private sector lobbyists Science outreach to public
8. Stakeholders are not valued, considered, or opposed by interventions	Better incentives for academics to focus on policy/practice relevant research Better stakeholder outreach in projects and inclusion of stakeholders in project design Include industry and private sector in research More integrated projects to move beyond just conservation outcomes Work with stakeholders from start of project
9. Scientists do not understand how policy is made	Better incentives for academics to focus on policy/practice relevant research Improve policy education of young scientists/scientists (e.g., through job shadowing, graduate training) More collaboration between scientists and policy‐makers (e.g., meetings, seminars, projects) Tailor evidence to audience–e.g., blogs, summaries, simple language, open access, policy briefs, infographics
10. Bad communication between scientists and policy‐makers	Better incentives for academics to focus on policy/practice relevant research Journals to translate key results into different languages More collaboration between scientists and policy‐makers (e.g., meetings, seminars, projects) More knowledge brokers (individuals to bridge the gap between science and policy) and system for it Tailor evidence to audience—e.g., blogs, summaries, simple language, open access, policy briefs, infographics

## 4 RESULTS

### 4.1 Phase 1 survey–compilation of top ten barriers and associated solutions

In the phase 1 survey, 32 barriers were proposed by 134 respondents (Table S4). From these responses, the top ten barriers and associated solutions (Table 1) were identified and used in phase 2.

### 4.2 Phase 2–Online survey

**Figure 1:**
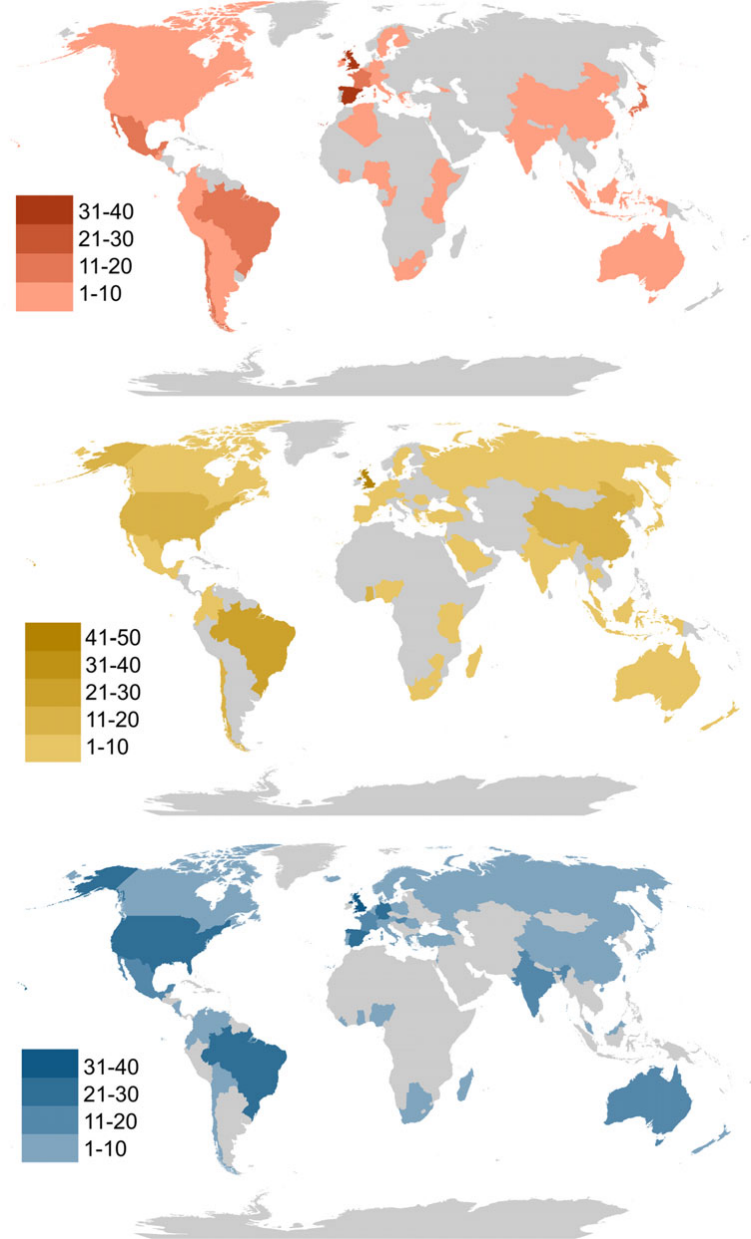
Heat map of responses by role (Red: Policy position, Yellow: Practitioners, Blue: Research Scientists)

The phase 2 quantitative survey was filled in by 758 people from 68 countries, comprising those in policy positions (238), practitioners (237), and research scientists (283) (Figure 1).

**Figure 2:**
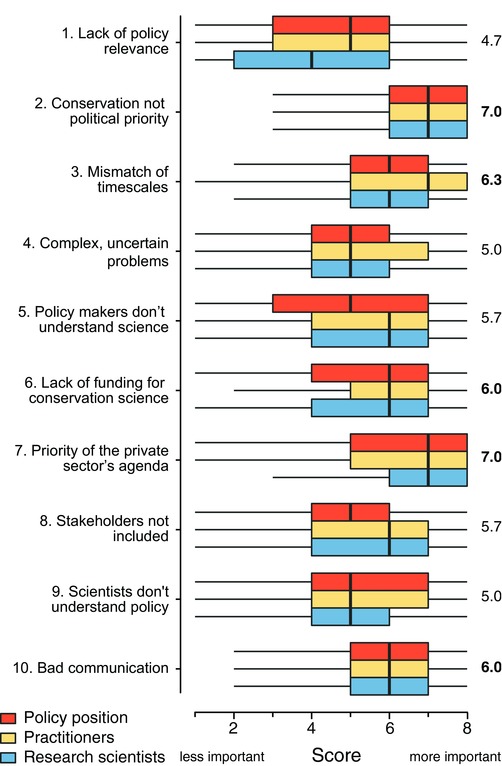
Boxplot (median, quartiles, and 5th/95th percentiles) showing the scoring for ten barriers restricting the use of conservation science in policy by three groups of conservation professionals. Numbers denote mean of medians across professionals. Bold numbers denote the top five ranked barriers

Based on the mean of median scores across the three roles, two barriers (2. Conservation not a political priority and 7. Priority of the private sector's agenda over conservation^3^) were given the highest importance (mean of medians = 7.0), followed by three barriers (mean of medians = 6.0–6.3; 3. Mismatch of timescales, 6. Lack of funding for conservation science and 10. Bad communication between scientists and policy‐makers). The other five barriers showed mean scores smaller than six (mean of medians = 4.7–5.7) (see Figure 2).

### 4.3 Understanding what explains barriers and solutions between science and policy

Scores provided by the 758 respondents varied significantly among both barriers and the three groups’ roles (Table 2). Though the interaction between barriers and role was significant; the majority of model deviance (79.2%) was accounted for by barrier identity (95.1% of the explained deviance), with role identity or the interaction term (role x barrier) giving negligible contributions (3.8%, Table 2). This suggests that patterns in scoring barriers were similar amongst roles. Patterns for barriers were reasonably consistent amongst countries with different Human Development Index levels, although there were variations (Figure S2).

**Table 2:** Total deviance (%) explained by the cumulative link models (rows) and percentage of the explained deviance accounted by factors “Barriers”/“Solutions,” “Role” and their interactive effect

		Percentage of the explained deviance		
Models	Explained deviance (%)	Barrier/Solution	Role	Barrier/Solution × Role
Barriers	79.2	95.1 (***)	1.2 (**)	3.8 (*)
Solutions for B2	74.9	73.7 (***)	16.3 (***)	10.1 (**)
Solutions for B3	76.5	91.1 (***)	6.7 (***)	2.2 (ns)
Solutions for B6	53.5	91.3 (***)	2.4 (ns)	6.4 (ns)
Solutions for B7	64.4	80.8 (***)	8.6 (***)	10.5 (*)
Solutions for B10	82.7	95.3 (***)	1.4 (*)	3.3 (*)

The significance of the effects shown in parentheses (ns: nonsignificant; ^*^ *P* < 0.05; ^**^ *P* < 0.01; ^***^ *P* < 0.001).

Scores of solutions to the top five barriers (barrier mean of medians ≥6) varied significantly and accounted for over 70% of the deviance explained by the models (Table 2). Scores for solutions varied significantly among roles in four out of the five barriers, and the interaction “solution × role” was significant in three out of the top five barriers. Yet, both role identity and the interaction term explained a much smaller proportion of deviance compared to the effect of solution identities (Table 2). This again shows that patterns in scoring solutions were similar among the three roles.

**Figure 3:**
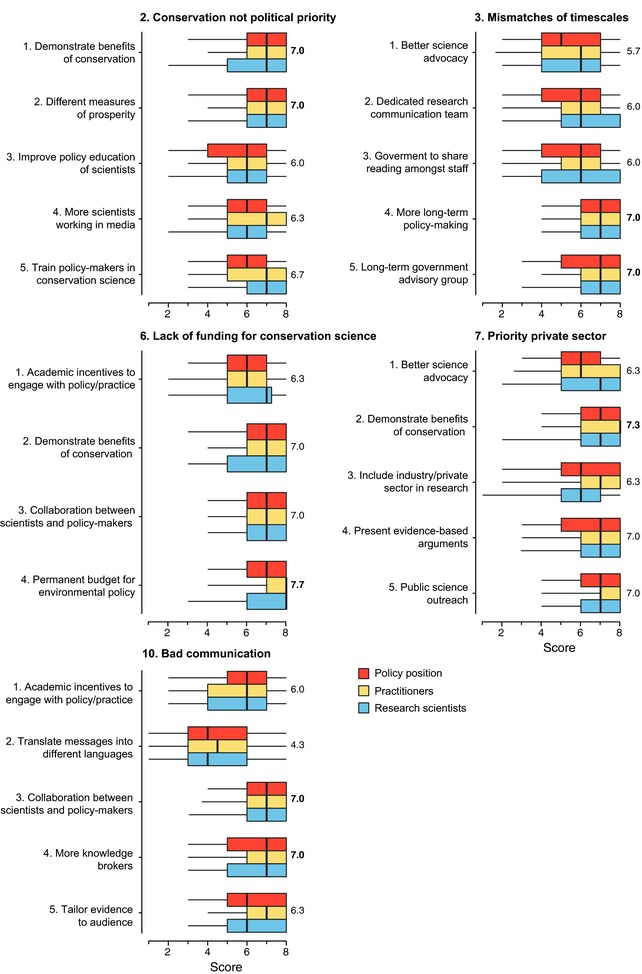
Boxplot (median, quartiles, and 5th/95th percentiles) showing the scoring for the solutions to the top five ranked barriers by three groups of conservation professionals. Numbers denote mean of medians across professionals. Bold numbers denote the highest ranked solution(s) for each barrier

Top‐ranked solutions for four of the barriers (2, 3, 6, 7) referred to the need to mainstream conservation, and to change the attitudes of policy‐makers in favor of proenvironmental, long‐term decision‐making; these included the need to develop “different measures of prosperity than GDP” (Barrier 2), the importance of “demonstrating the benefits of conservation” (Barriers 2, 7), and a dedication to “encouraging the strategic use of science for long‐term policy‐making” (Barrier 3) with associated “long‐term government advisory groups” (Barrier 3) and a “permanent environmental budget” (Barrier 6). In response to Barrier 10 (“bad communication between scientists and policy‐makers”), the solutions “more knowledge brokers” and “collaboration between scientists and policy‐makers” were ranked highly (Figure 3).

**Figure 4:**
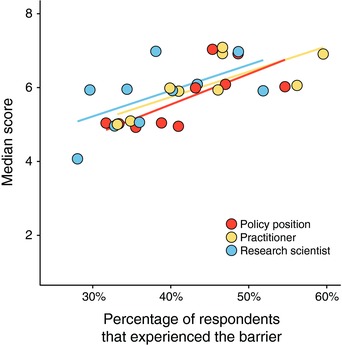
Relationship between the percentage of respondents that experienced a barrier and the median barrier score for each of the three professional groups. For illustrative purposes only, regression lines are shown

Participants were also asked whether they had experienced any of the ten barriers. Overall, we found a consistent positive correlation across roles between experiencing a barrier and ranking it more highly (Kendall's *τ* = 0.49–0.77, all *P* < 0.033—see Figure 4). The top five most experienced barriers were the top five ranked barriers, although the order varied (Table S5 and Figure S3).

## 5 DISCUSSION

### 5.1 A surprising amount of agreement?

A logical conclusion from previous research (e.g., Arlettaz et al., 2010) would be that policy‐makers, practitioners, and scientists disagree on the barriers and solutions to the use of conservation science in policy. In reading the exchange between Sutherland, Spiegelhalter, and Burgman (2013) and Tyler (2013), for example, we may have expected scientists to place the emphasis on training policy‐makers to comprehend science, in other words blaming policy‐makers for lack of understanding, rather than criticizing themselves for communicating evidence badly (see Kenny, Rose, Hobbs, Tyler, & Blackstock, 2017). Contrastingly, one may have expected policy‐makers to focus on encouraging scientists to present their evidence in a user‐friendly manner, instead of blaming themselves for lack of understanding. Yet, our results suggest that there is, in fact, widespread agreement, and thus, at the very least, that disagreement between groups would not be the limiting factor preventing the successful uptake of highly ranked solutions. Our results also suggest that Sutherland and Wordley's (2017) notion of “evidence complacency” is not caused by a lack of awareness of science on the part of decision‐makers; rather, their use of evidence may be constrained by other drivers, such as political barriers.

Our results suggest that there is little difference between rankings of barriers and solutions amongst different genders, and individuals with greater or less experience in conservation (Figures S4–S6). In addition, there is little difference between rankings provided by individuals in different countries ranked in order of Human Development Index (Figure S2), although poorer countries did prioritize “lack of funding for conservation science” more highly.^4^

It is interesting to note that the two top‐ranked barriers (2 and 7) relating to the low priority of conservation were not the most experienced (although they were in the top‐five for “experienced” too). This suggests that they are perceived to be *the* major barriers, even by those not directly experiencing them. Other highly ranked barriers were the most experienced, which suggests that respondents were ranking them based on real‐life exposure rather than merely perception.

### 5.2 Barriers

Here, we examine the top five barriers, offering a selection of quotations written by online survey respondents in the “other” category (S5 for discussion of barriers 6–10).

Three of the five top‐ranked barriers relate in some way to the low priority of conservation on the policy agenda – “conservation not a political priority,” “priority of the private sector's agenda over conservation,” and the “lack of funding for conservation science.” While opinion polls have suggested that the environment is an important issue (EU Barometer, 2014), it is rarely selected as the top priority (Marshall et al., 2017), which in turn influences the agenda of policy‐makers. An extract from one survey highlights this (see Q1‐2 S6 for more): “If you do not have public support for conservation, you will rarely gain political support” (Policy position, Ireland).

Research suggests that anti‐environmental lobbying of some private sector groups convinces policy‐makers to put industry needs ahead of conservation (Guerrette, 1986). As one practitioner from Brazil noted, “conservation is effective when there are no economic interests.” Where the private sector has attempted to embrace an environmentalist agenda, there have been claims that nature is exploited (Rodriguez‐de‐Francisco & Budds, 2015).

“Lack of funding for conservation science” was also ranked in the top five barriers. Gill et al. (2017) found that the effectiveness of MPAs was influenced most by staffing and resources, yet there are finite resources for experimentation, implementation, and monitoring (Sutherland, Shackelford, Rose, 2017). Our study noted that this was a particular problem in poorer countries (Figure S2).

A contributory factor to conservation not being a political priority is the “mismatch of timescales.” Policy‐makers usually focus on short‐term issues (Lawton, 2007), and demand evidence quickly. Conservation science often takes a longer‐term view with slower reporting timescales. Since conservation is a long‐term issue, relevant policies are easily “kicked into the long grass” when other short‐term needs arise. Furthermore, scientists rarely seize upon policy windows for the uptake of knowledge (Rose et al., 2017).

The final barrier in the top five related to “bad communication between scientists and policy‐makers.” Poor communication, and lack of interaction between these groups, manifests itself in a variety of ways, including lack of access to scientific articles, inadequately communicated policy/management demands, and conservation science being presented in unusable formats (Marshall et al., 2017; Walsh, Dicks, & Sutherland, 2015). Although there is some overlap between science and policy/practice spheres (Rose, 2014; Vadrot, 2014), they are distinct. Fundamental differences in workflows, background, and objectives create challenges for successful communication (Farwig et al., 2017). A survey respondent suggested that it was an “illusion” to think that effective joint meetings and seminars could be held with scientists and policy‐makers because of different workflows (Policy position, Germany).

### 5.3 Solutions

Increasing the priority of conservation in public policy would seem to be the key issue as agreed by all groups (Figure 3). A staff member in a policy position (Germany) stated that “compiling more scientific facts does not help” (also Q3‐4 S6). Instead, several comments wanted a “revolution” in societal attitudes (Q5‐7 S6). Establishing a long‐term mind set to environmental policy, including setting up advisory bodies that span political timescales, was considered necessary. Given the short‐term nature of politics (Lawton, 2007), it is challenging to consider that adopting different measures of prosperity can occur without a step‐change in voting. As one survey respondent noted, “if the electorate are not interested in long‐term solutions, policy‐makers will not be” (Policy position, United Kingdom).

To foster a long‐term positive view of the environment, “raising awareness among the public and decision‐makers regarding the long‐term consequences of inaction” (Policy position, Switzerland) was considered important. Two highly ranked solutions for “conservation not a political priority” and “priority of the private sector's agenda over conservation” suggested better public outreach to show the benefits of conservation. The “paradox of timescales” (Lawton, 2007) could be overcome if policy‐makers were elected on the strength of their long‐term environmental commitment. As one respondent in a U.K. policy position stated, “shifting policy means shifting the politics, which is only possible if one shifts public opinion” (also Q8 S6).

The overwhelming message for overcoming the top‐ranked barriers, therefore, is to convince policy‐makers to adopt pro‐environmental long‐term policies, and to measure prosperity in other ways than just GDP. This requires larger numbers of people to join the conservation community and demand convincing, inclusive messages (Begon, 2017). We stress the need for several messages to be told since each person responds differently to different messages (Blicharska & Grandin, 2015). Telling good news stories might help (Balmford & Knowlton, 2017), as people need to be inspired, rather than served with doomful scenarios (https://conservationoptimism.com). It is also vital to know how to change behavior (Tannenbaum, Fox, & Rogers, 2017). Also it is worth remembering that policy‐makers are people too and they can be influenced by relevant, human‐based stories (Begon, 2017); a fact noted by a practitioner from Brazil who urged conservationists to make the problem “more real” by developing closer relationships with policy‐makers. Conservationists could frame carefully for nature conservation (Mace, 2014), as varied arguments may be more convincing to different people at different times (Tinch et al., 2016).

Our results suggest that recent calls for science to become more inclusive of society may be warranted (Colloff et al., 2017; Keeler et al., 2017; Nature Human Behaviour, 2017; Redford et al., 2015). A practitioner from Uganda argued that ‘it is necessary to win the hearts and minds of people’, recruiting them to the conservation cause, in order to convince policy‐makers that it is a priority issue. The same practitioner thought that this had been “downplayed” in previous conservation efforts, and a respondent from Italy (policy position) argued that conservationists have wrongly focused on “addressing already acquired audiences.” Our work also suggests that there may be a need to involve the private sector more as allies of conservation.

To improve communication between scientists and policy‐makers, two solutions related to better collaboration and the use of knowledge brokers scored “7.” Research scientists could be encouraged to collaborate with policy‐makers through better reward systems, and to respond quickly to evidence demands (Neßhöver et al., 2016). Policy‐makers could likewise be encouraged to work closely with the research community and make demands for evidence available to researchers. Where collaboration is not possible, knowledge brokers are vital. They speak the language of both science and policy and are important entrepreneurs linking the two worlds (Cvitanovic et al., 2015; Nguyen, Young, & Cooke, 2017). Scientists could make more use of key intermediaries, for example, policy think‐tanks and NGOs, who may have direct lines into public, business, or policy‐makers, links that are difficult for universities and academics to develop. More support is required to create, and appreciate, knowledge brokers and this requires a shift towards valuing cross‐disciplinarity.

### 5.4 Evaluation

The major positive of this study is that the survey was translated into multiple languages and responded to by different types of respondents globally. There were, of course, some flaws to the methodology. These included respondents providing information on their perceptions of the barriers and solutions. However, we counteracted this by asking respondents if they had experienced the barriers; the fact that the highly ranked barriers were also the most experienced suggests that responses were based on real‐life exposure. Also, although we may have expected individual groups to blame failings on the part of others, the fact that we found widespread agreement seems to suggest that this was not a major problem.

## 6 CONCLUDING REMARKS

Contrary to previous research that highlights disagreement between scientists and decision‐makers, we found that people in policy positions, practitioners, and research scientists across countries tended to agree on the barriers and solutions to incorporating conservation science in policy. In order to overcome highly ranked barriers related to the low priority of conservation in public policy, top solutions focused on the need to mainstream conservation. The ranking of solutions suggests that harnessing public (and policy) support for a pro‐environmental, long‐term approach to decision‐making can improve the prospects for evidence‐informed conservation policy. Our study thus suggests we need to appreciate the importance of winning the hearts and minds of people to help us achieve evidence‐informed conservation policy. The study also suggested that there might be small variations in the priority of barriers and solutions in different contexts, for example, poorer countries considered “lack of funding for conservation science” to be a particular concern (although the differences were small). This illustrates the importance of understanding national and regional contexts for science‐policy interactions.

The optimistic message from this study relates to the apparent agreement between research scientists, policy‐makers, and practitioners about the key barriers and solutions to the use of conservation science in policy. We argue, therefore, that it should be possible to implement solutions to win the hearts and minds of people.

## Supporting information


**FIGURE S1** Flow diagram illustrating the survey methodology
**FIGURE S2** Ranking of barriers by role according to Human Development Index
**FIGURE S3** Proportion of different roles (Red: Policy position, Yellow: practitioners, Blue: Policy position) experiencing the barriers
**FIGURE S4** Proportion of male and female respondents to the online survey by role
**FIGURE S5** Ranking of barriers by gender
**FIGURE S6** Years of experience of online survey respondents by role
**TABLE S1** Process followed for categorizing roles
**TABLE S2** List of phase one survey respondents by role, years of experience, and country of work
**TABLE S3** Ranking of barriers and solutions from phase one survey
**TABLE S4** All barriers mentioned in the phase one survey
**TABLE S5** (a‐top) Ranking of most experienced barriers, and (b‐bottom) Overall barrier rank versus ranking of how highly experienced it was

Translated Abstract

## Footnotes

1 See supplementary material (Table S1) for information on how we categorised respondents. Briefly, people in policy positions were generally either politicians, civil servants (including scientists and economists working for government or a statutory agency), or NGO staff who had a specific remit for policy work, and hence for interacting with policy communities. Practitioners were comprised of roles that implemented conservation on the ground, whereas research scientists were post‐docs or academics in university or research institutions, or those with a specific research remit in an NGO. We acknowledge that some people had dual roles which could have overlapped, but we asked respondents to pick the role that best suited their primary job.

2 This total figure included 53 academics/research scientists, 33 people in policy positions, and 21 practitioners. 24 students also responded, but responses from this group were lower for the second online survey, and thus their responses are not included in the final analyses (see Table S2). The other three respondents were publishers (2) or a knowledge broker (1).

3 We acknowledge that these barriers are interlinked, in the same way for example, as lack of funding for conservation science is linked to lack of political priority. However, we argue that they were sufficiently different to include as separate barriers, particularly since barrier 7 specifically identified the power of the private sector to override environmental arguments.

4 There were subtle variations in ranking of barriers and solutions by HDI (Figure S2). A “lack of funding for conservation science” was ranked more highly in groups of countries with low HDI, mainly across Africa and South America. This would suggest that adequate funding for conservation science is a particularly acute problem in countries where financial resources are low. The barrier of not “including or valuing stakeholders” in conservation science also tended to be scored more highly in countries with low HDI. This might perhaps be linked to the low resources for outreach.
