# Supplementary material for: The major barriers to evidence‐informed conservation policy and possible solutions
Source: Conserv Lett. 2018 May 8;11(5):e12564. doi: 10.1111/conl.12564 (PMC6473637; doi:10.1111/conl.12564)
Supplement: Supplementary file 1 — FIGURE S1 Flow diagram illustrating the survey methodology FIGURE S2 Ranking of barriers by role according to Human Development Index FIGURE S3 Proportion of different roles (Red: Policy position, Yellow: practitioners, Blue: Policy position) experiencing the barriers FIGURE S4 Proportion of male and female respondents to the online survey by role FIGURE S5 Ranking of barriers by gender FIGURE S6 Years of experience of online survey respondents by role TABLE S1 Process followed for categorizing roles TABLE S2 List of phase one survey respondents by role, years of experience, and country of work TABLE S3 Ranking of barriers and solutions from phase one survey TABLE S4 All barriers mentioned in the phase one survey TABLE S5 (a‐top) Ranking of most experienced barriers, and (b‐bottom) Overall barrier rank versus ranking of how highly experienced it was [file CONL-11-na-s001.docx]

**The major barriers to evidence-informed conservation policy and possible solutions**

**Supplementary material**

Table of Contents

S1: Supplementary methodology3

S2: Phase one survey8

S3: Explanation of codes used in phase one9

S4: Phase two survey11

S5: Barriers 6-10 discussion19

S6: Supporting quotes20

List of figures

Figure S1: Flow diagram illustrating the survey methodology……………………………………………21

Figure S2: Ranking of barriers by role according to Human Development Index………………………..23

Figure S3: Proportion of different roles experiencing the barriers………………………………………..23

Figure S4: Proportion of male and female respondents to the online survey by role……………………..24

Figure S5: Ranking of barriers by gender…………………………………………………………………25

Figure S6: Years of experience of online survey respondents by role……………………………………26

List of tables

Table S1: Process followed for categorising roles………………………………………………………...27

Table S2: List of phase one survey respondents by role, years of experience, and country of work……..28

Table S3: Ranking of barriers and solutions from phase one survey……………………………………...29

Table S4: All barriers mentioned in the phase one survey………………………………………………..30

Tables S5 (a) Ranking of most experienced barriers and (b) Overall barrier rank versus ranking of how highly experienced it was……………………….........................................................................................31

**S1 Supplementary methodology**

Figure S1 illustrates the flow of the surveys used in this study. Ethical approval was gained for this study through standard institutional procedures, which involved scrutiny by the ethics board at the Department of Geography (Cambridge). Each iteration of the survey included clear instructions about the purpose of the study, data storage, anonymity, and details about how the data would be used. The survey was optional and the participant could withdraw at any time.

*Phase 1 scoping survey questions*

In the phase 1 scoping survey [S2], respondents were asked to i) select a role description, ii) name up to three barriers preventing the use of conservation science in policy-making, and iii) suggest solutions for the proposed barriers. The barriers and solutions sections were left open-ended such that the respondents were not constrained by our beliefs. Additional information asked of respondents in the second stage of scoping included job description, number of years of experience, gender, and main country of work. These variables enabled further analysis of whether perceptions of conservation science-policy interfaces differed between different segments. In the scoping surveys, several role categories were available for selection in the surveys (e.g. research scientist, policy-maker, practitioner, student, and a box for ‘other’ roles).

*Scoping survey dissemination*

The first iteration of Phase 1 targeted delegates at a science-policy conference (British Ecological Society/Cambridge Conservation Initiative conference, April 2016). These respondents were mainly academics from the UK, with fewer respondents from policy and practice spheres. A second iteration was used to address this bias, extending the number and range of respondents. Respondents to the second iteration included (see Table S1 for categorisation of respondents to both iterations):

1. Academics, policy makers and practitioners working on, or with, the EU Biodiversity Observation Network Project,
2. People in policy positions within the Department for Environment, Food and Rural Affairs, Natural England/Environment Agency (UK government department and statutory agencies responsible for conservation/environment), and European agencies (e.g. European Environment Agency),
3. NGOs based within the David Attenborough Building in Cambridge (Birdlife International, United Nations Environment Programme-World Conservation Monitoring Centre, Royal Society for the Protection of Birds, British Trust for Ornithology, Traffic, Tropical Biology Association, Flora and Fauna International, International Union for the Conservation of Nature) with an instruction to distribute it to staff networks around the world. Cambridge Conservation Forum, a network linking practitioners and researchers, were also involved,
4. Conservation practitioners from Africa and South America based on key informants in the Cambridge Conservation Initiative.

In total, 134 responses were gained^[[1]](#footnote-1)^ from 30 countries [Table S2] (47 from the conference, 87 from wider networks).

*Phase one analysis and phase two survey*

Responses for both barriers and solutions were coded [S3], and a list of the top ten barriers was created on a quantitative basis [Table S3]. Owing to the disproportionate number of respondents from the Global North in the scoping surveys, it is possible that the outcomes might have been biased. To overcome this bias, we divided the responses into Global North and Global South (based on the Brandt line). We weighted the responses based on the proportion of responses and recalculated the ranking for the top ten barriers. However, the calibration did not change the barriers in the top ten. Therefore, we continued with the top ten barriers and corresponding solutions for the online survey [Table S3].

We asked respondents to the phase 2 survey [S4] to rank the top ten barriers on a Likert Scale of 1 (low)-8 (high), and ensured that we did not present the barriers in exactly the same order to their original ranking from the scoping survey. Not all solutions from Phase 1 were included in the Phase 2 survey. Instead, the solutions from the first survey were ranked by majority votes. A solution was included in the online survey if it was mentioned four times or more, or if the solution was unique across all barriers (i.e. not reported for any of the other barriers in the top ten list). They were similarly ranked on the same scale. The respondents were also given the option to add to the list of barriers and solutions. Other information asked for included role, gender, country where they worked most often (we accepted only the first country), years of experience in conservation], which were used to test their effects on scoring patterns (see *Statistical analysis* for more detail), and whether they had experienced any of the listed barriers in practice.

There is evidence to suggest that language is a critical barrier when compiling information globally (Amano *et al.*, 2016), and many previous studies on conservation science-policy interfaces have been conducted in English. This can prevent many actors in the global, and diverse, conservation community from being involved in academic research. To address this bias (particularly for countries in which English is not the first language), we translated the survey into five different languages (Spanish, French, Portuguese, Chinese and Japanese). An online version of the survey was designed using Google forms for all the languages except Chinese. The Chinese version was designed using a Chinese online survey platform called Tencent questionnaires (<https://wj.qq.com/)>. The online version of the English survey was launched on 11^th^ November 2016 while the other language versions were launched by 29^th^ November 2016. A range of different approaches were used to disseminate the survey as widely as possible (e.g. known networks, social media, email lists). All survey links were live for approximately two months. No incentive was offered to fill in either survey, and ethics clearance was sought before conducting the surveys.

Based on the number of responses, and the desire to ensure that multiple perspectives of conservation science-policy interfaces were harnessed, we focused on three distinct roles in analysis of the online survey: (*i*) people in policy positions (e.g. policy-makers and scientists in policy departments), (*ii*) practitioners (e.g. NGO staff with a practice mandate, reserve manager), and (*iii*) research scientists (e.g. postdoctoral and other research scientists, not students). Other additional categories were either excluded from the analysis (e.g. students), or merged into other categories [Table S1]. We include a caveat here to say there is fluidity and overlap between these roles, but we placed respondents in the final category to which we felt they were best suited. The broad category of ‘people in policy positions’ encompasses policy-makers, scientists and other civil servants working in government (or in government agencies), and NGO staff working in an organisation with a policy mandate.

*Statistical analysis*

Statistical analysis of the phase 2 quantitative survey focused on scores given by people in policy positions, practitioners, and research scientists (Likert scale of 1 [not important] to 8 [very important]). Cumulative link models were applied to test the relationship between the score of each barrier/solution (as ordinal response variables) and two explanatory variables, barrier/solution identity and the role of respondents (policy position/practitioners/research scientists), as well as their interaction. The significance level of each term was derived from likelihood ratio tests and deviance for each term was also calculated, following Christensen (2015a). To rank the overall importance among distinct barriers and solutions, we calculated the mean of the median scores across the three roles for each barrier/solution. The aim of using the mean of medians, instead of the overall median per barrier/solutions was to control for the difference in the sample size across the different roles.

We used the Kendall’s rank correlation coefficient (τ) to test – in each of the three studied roles – for positive relationships between the percentage of respondents that experienced each barrier and the median barrier score. We thus performed one-tailed tests because we expected these relationships to be positive.

*Sensitivity analysis*

Patterns in scoring can also be affected by other covariates. We therefore explored the sensitivity of results from the analysis described above to three covariates: the gender of respondents, years of experience in conservation, and the Human Development Index (2015 UNDP figures) of the country where they worked most often.

The gender of respondents was significantly different among roles (Chi-square = 14.2, p < 0.001) and policy position had a particularly high proportion of males (66.5%) as compared to that proportion among practitioners (51.3%) and research scientists (52.1%) (Figure S4). Nevertheless, when testing effects of barrier/solution identities, the gender of respondents and their interactions on scores provided by policy position, the interaction term was significant neither for barriers (p = 0.976) nor for solutions to nine out of the ten barriers (p = 0.157–0.645 for solutions to all barriers except to Barrier III). Even for solutions to Barrier III, where the interaction term was significant (p = 0.045), the interaction term only explained 7% of the deviance explained by the model (69%). This result indicates that patterns in scoring different barriers and solutions were similar between genders in policy position and thus, we concluded that the effect of the gender imbalance in policy position was almost negligible (Figure S5). Years of experience in conservation were also significantly different among roles (ANOVA after square-root transformation: *F*_2,753_= 4.78, p = 0.009), however, 'role' only accounted for 1% of variance in years of experience (R^2^ = 0.01) and the difference among roles was very small (maximum of 9.8% difference; Figure S6). Thus we decided not to account for the effect of years of experience in the analysis.

To evaluate if scoring patterns depended on the socioeconomic/geopolitical circumstances of the countries where respondents worked most often, we compared the top-scored barriers (based on the mean of median scores by the three roles) among four categories of Human Development Index (HDI), into which all countries were categorized: ‘low’, ‘mid-low’, ‘mid-high’ and ‘high’. We established the range of HDI values for each category by calculating the median and quartiles across all the respondents (< Q_25_; Q_25_–median; median–Q_75_; > Q_75_). Top-scored barriers were consistent across HDI categories; either of the two barriers with the highest overall scores also received the highest median score in the four HDI groups (Figure S2). Also, the top-five barriers were the same across HDI groups. In fact, we found significant rank correlations between all pairs of HDI groups in their scoring patterns at the barrier level (Kendall’s τ = 0.58–0.86, all P < 0.012). Therefore, we concluded that the scoring patterns reported in the result section were consistent across regions with representing a huge range of socioeconomic/geopolitical scenarios (Figure S2).

The analysis was conducted in R (R Core Team 2016) and cumulative link models were implemented with the R package ordinal (Christensen 2015b). The analysis showed that biases in gender, experience, and Human Development Index, did not seem to affect significantly the ranking of barriers and solutions in this study.

**S2 Phase one survey**

We would be grateful if you could fill in this short survey to help us with our work. We are interested in barriers/solutions to improving the use of conservation science in policy-making. If you have any questions please email Dr David Rose at [*******](mailto:dcr31@cam.ac.uk) All answers are anonymous (answers not stored in a way allowing link with email addresses) – please email back your completed surveys by *** to *******

1. **Which of the following roles best describes your position? (circle)**

- Academic (please state discipline)
- Scientist (in statutory agency i.e. linked to government)
- NGO staff member (practice-focused)
- NGO staff member (policy-focused)
- Policy-maker (government or statutory organisation, i.e. linked to government)
- Policy-maker (not linked to government)
- Other (please state): .............................................

1. **In your view, what role do you think that scientists should play in the policy-making process in conservation? (one sentence)**

**3. In your view what are the main barriers to the use of conservation science in policy? (list up to three)**

1. **For the barriers listed, list your proposed solutions.**

Second iteration (additional questions)

1. **How many years have you been involved in doing science or informing/making policy decisions about nature conservation?**
2. **What country do you have the most experience of working in?**

**S3 Explanation of codes used in phase one**

1. **Lack of policy relevant science**: any response covering the mismatch between conservation science produced by scientists and policy demands. This code included comments such as ‘results don’t match policy or practice priorities’ and ‘academics focus on funding not policy/practice relevant study’.
2. **Conservation not a political priority:** any comment saying that conservation/conservation science were not weighted highly enough in policy terms; in other words, other priorities were pursued that might contradict conservation science. Comments included in this code included ‘other issues like the economy prioritise’, ‘conservation cannot compete with other priorities’, ‘lack of appreciation of the importance of conservation’, ‘absence of political will for conservation’, and ‘policy-makers not interested in conservation’.
3. **Mismatch of timescales**: any comment relating to the mismatch between the timescales of producing conservation science, the temporal nature of required conservation interventions, and policy timescales. Comments merged into this code included themes related to (1) the time taken to produce science versus policy timescales e.g. ‘scientists take too long to report findings’, ‘policy-makers need science quickly and it isn’t always available’, and ‘windows of opportunity are missed by scientists’, and (2) the mismatch between short-term political priorities versus long-term environmental policies e.g. ‘conservation demands long-term decision-making which policy-makers don’t like’, ‘policy-makers are too focused on the short-term’, ‘thinking of future generations is unpopular’, and ‘short term vote winning is more important’.
4. **Complex, uncertain problems**: any comments relating to the uncertainty or complexity of conservation scientists, which make it difficult for policy-makers to know what to do. Comments merged into this category included ‘lack of evidence availability’, ‘uncertain and unpredictable futures’, ‘lack of scalable conservation data’, and ‘lack of monitoring data’.
5. **Policy-makers don’t understand science:** any comment relating to the problem of policy-makers interpreting conservation science. Comments merged into this category included those such as ‘policy-makers are not trained in science’, ‘policy-makers are not scientists’, and ‘conservation science hard to understand for non-specialists’.
6. **Lack of funding for science**: any comment relating to the limited funding for conservation science. Comments merged into this code included ‘lack of resources for monitoring’, ‘lack of resources for science’, ‘lack of policy relevant science funding’, and ‘cost of science’.
7. **Priority of the private sector’s agenda over conservation:** any comment related to the perceived greater influence of the private sector over conservation needs. Comments merged into this code included ‘private sector dominate over conservation needs’, ‘power of private sector lobbying’, and ‘private sector wields power in policy-making’.
8. **Stakeholders are not valued, considered, or are opposed by interventions:** any comment related to the conflict between conservation science and stakeholders. This could include the fact that stakeholders are not valued or included in projects, e.g. ‘not enough compensation for local stakeholders’, ‘no training for conservation scientists to work with local people’, and ‘lack of diversity of views in recommendations’. Also included were comments where conservation was challenged by stakeholders, including ‘polarised, divergent stakeholder views’ and ‘conflicts between stakeholders’.
9. **Scientists don’t understand how policy is made:** any comment related to a lack of understanding of policy on the part of scientists. Comments merged into this code included ‘scientists don’t understand the policy-making process’ and ‘scientists don’t know about policy and can be too pompous’.
10. **Bad communication between scientists and policy-makers**: any response addressing the problems of communicating conservation science and/or policy demands between scientists and policy-makers. Also included in this code were comments relating to the lack of/poor interaction between scientists and policy-makers which were thought to exacerbate communication problems. Initial codes merged into this category included; ‘terminology not understood by different actors’, ‘evidence disseminated in poor formats such as non-open access journals’, ‘poor interaction between scientists and policy-makers’, ‘no collaboration between scientists and policy-makers’, and ‘communication gap between science and policy’.

**Solutions were also coded into larger categories through a similar process of initial coding, then they were merged into larger categories shown in Table 1.**

**S4 Phase two survey**

Note: the word science is used to mean knowledge and ideas from the natural sciences, social sciences, humanities, and lay knowledge.

* Required

1. Which of the following roles best describes your position? * *Mark only one oval.*

Academic (including post docs and research associates)

Student

Scientist (in statutory agency i.e. linked to government)

Practitioner (government)

Practitioner (NGO)

Policy-maker (government)

Policy-maker (NGO)

Other:

2. Please state the country you work in. *

3. Please state the number of years you have been involved in conservation (either in doing science or in forming/making policy decisions about nature conservation or both). *

<1 1 2 3 4 5 6 7 8 9 10 11 12 13 14 15 16 17 18 19 20 21 22 23 24 25 26 27 28 29 30 >30

4. Please state your gender identity below.

Female Male Rather not say Other:

**Barriers to the use of conservation science in the science- policy interface**

5. Please score the barriers by importance i.e. the degree to which you think the barrier prevents the use of conservation science in policy-making (1=lowest importance, 8=highest importance).

*1. Lack of policy relevant science (i.e. conservation science lacks real world application)*

*2. Conservation not a political priority*

*3. Mismatch of timescales (i.e. policy-makers focus on short-term instead of long-term; science may not be able to report quickly and seize short-term opportunities)*

*4. Complex, uncertain problems (i.e. science is uncertain or there is a lack of information)*

*5. Policy-makers don't understand the scientific evidence communicated*

*6. Lack of funding for conservation science*

*7. Priority of the private sector’s agenda over conservation (i.e. private sector are powerful lobbyists who can convince policy- makers to back them against conservation)*

*8. Stakeholders are not valued, considered, or opposed by interventions (i.e. stakeholders not included in scientific problems leading to misunderstanding and conflicts)*

*9. Scientists don't understand how policy is made*

*10. Bad communication between scientists and policy-makers (including lack of interaction between them)*

6. Have you personally experienced any of these barriers? If so, which ones? (Please mention the barrier numbers noted above).

7. Have you perceived any other barriers not listed above? If so, please mention them here (100 words max).

**Linking barriers to solutions (order changed to minimise chances of bias)**

Please score the solutions for each of the barriers (1= lowest importance, 8= highest importance).

**Barrier 1. Bad communication between scientists and policy-makers**

Please score the solutions (1= low importance, 8= highest importance).

*Mark only one oval per row.*

Better incentives for academics to focus on policy/practice relevant research Journals to translate key results into different languages

More collaboration between scientists and policy-makers (e.g. meetings, seminars, projects)

More knowledge brokers (individuals to bridge the gap between science and policy) and system for it

Tailor evidence to audience - e.g. blogs, summaries, simple language, open access, policy briefs, infographics

**Barrier 2. Lack of policy relevant science**

Please score the solutions (1= low importance, 8= highest importance).

*Mark only one oval per row.*

Ask policy relevant questions from start of project, including policy- makers

Better incentives for academics to focus on policy/practice relevant research

Embed young scientists in the field and train them on importance of real world science application

Improve policy education of young scientists/scientists (e.g. through job shadowing, graduate training)

More collaboration between scientists and policy-makers (e.g. meetings, seminars, projects)

Please mention other possible solutions to this barrier (100 words max).

**Barrier 3. Conservation is not a political priority**

Please score the solutions (1= low importance, 8= highest importance). *Mark only one oval per row.*

Demonstrate benefits of conservation (including economic value)

Develop different measures of prosperity than just GDP/economy

Improve policy education of young scientists/scientists (e.g. through job shadowing, graduate training)

More scientists working in/with media to engage policy-makers and public

Train policy-makers in conservation science to help them see the importance of conservation

Please mention other possible solutions to this barrier (100 words max).

**Barrier 4. Mismatch of timescales**

Please score the solutions (1= low importance, 8= highest importance).

*Mark only one oval per row.*

Better science advocacy from scientists

Dedicated office at research institutions to help researchers communicate key information

Encourage government departments to share reading of scientific outputs

Encourage the strategic use of science for long-term policy- making

Set up government advisory body that spans political timescales

Please mention other possible solutions to this barrier (100 words max).

**Barrier 5. Complex, uncertain problems**

Please score the solutions (1= low importance, 8= highest importance).

*Mark only one oval per row.*

Better communication of uncertainty

More transparency about uncertainty

Standardise methods and indicators for conservation to improve communication

Train scientists in a variety of communication skills

Transdisciplinary research to be encouraged

Please mention other possible solutions to this barrier (100 words max).

**Barrier 6. Policy-makers don't understand science**

Please score the solutions (1= low importance, 8= highest importance). *Mark only one oval per row.*

Better science education in schools and universities to improve science literacy of population

More knowledge brokers (individuals to bridge the gap between science and policy) and system for it

More scientists working in media to engage policy-makers and public

Tailor evidence to audience - e.g. blogs, summaries, simple language, open access, policy briefs, infographics

Train policy-makers in science

Please mention other possible solutions to this barrier (100 words max).

**Barrier 7. Lack of funding for science**

Please score the solutions (1= low importance, 8= highest importance).

*Mark only one oval per row.*

Better incentives for academics to focus on policy/practice relevant research

Demonstrate benefits of conservation (including economic value)

More collaboration between scientists and policy-makers (e.g. meetings, seminars, projects)

Permanent budget for environmental policy making

Please mention other possible solutions to this barrier (100 words max).

**Barrier 8. Priority of the private sector’s agenda over conservation**

Please score the solutions (1= low importance, 8= highest importance). *Mark only one oval per row.*

Better science advocacy

Demonstrate benefits of conservation (including economic value)

Include industry and private sector in research

Provide evidence-based argument to counter private sector lobbyists

Science outreach to public

Please mention other possible solutions to this barrier (100 words max).

**Barrier 9. Stakeholders are not valued, considered, or opposed by interventions**

Please score the solutions (1= low importance, 8= highest importance). *Mark only one oval per row.*

Better incentives for academics to focus on policy/practice relevant research

Better stakeholder outreach in projects and inclusion of stakeholders in project design

Include industry and private sector in research

More integrated projects to move beyond just conservation outcomes

Work with stakeholders from start of project

Please mention other possible solutions to this barrier (100 words max).

**Barrier 10. Scientists don't understand how policy is made**

**Please score the solutions (1= low importance, 8= highest importance).**

*Mark only one oval per row.*

Better incentives for academics to focus on policy/practice relevant research

Improve policy education of young scientists/scientists (e.g. through job shadowing, graduate training)

More collaboration between scientists and policy-makers (e.g. meetings, seminars, projects)

Tailor evidence to audience - e.g. blogs, summaries, simple language, open access, policy briefs, infographics

Please mention other possible solutions to this barrier (100 words max).

Please mention any comments or suggestions about this survey. (100 words max)

**S5 Barriers 6-10 discussion**

Other barriers in the top ten were ranked less highly (Barriers 1, 4, 5, 8, 9), including ‘policy-makers don’t understand science’ (B5) and ‘scientists don’t understand how policy is made’ (B9). Sutherland *et al.* (2013) and Tyler (2013) discussed these barriers, suggesting that the different skills associated with each role made it difficult to understand other workflows. Policy-makers are rarely scientists, and therefore would not be expected to understand fully the scientific method, whereas scientists are rarely given training in policy processes (Rose, 2015). Commenting on the lack of policy understanding from scientists, one practitioner from the UK stated in the online survey, ‘I often feel that the policy recommendations are written for a world where we are ruled by a benign dictator, rather than messy democracies’.

Limited communication between science and policy exacerbates the problem that research scientists are producing a ‘lack of policy-relevant science’ (B1), a theme noted in much research (e.g. Braunisch *et al.,* 2012; Mcnie, 2007; Milner-Guland *et al.,* 2009; Walsh *et al.,* 2015). It is interesting to note, however, that this was the least highly-ranked barrier of those presented in the online survey.

Predicting biodiversity loss, and the effects of conservation interventions, relate to the barrier of ‘complex, uncertain problems’ (B4) (Game *et al.,* 2014). Lack of funding for conservation science undoubtedly restricts the capacity of the conservation community to fill data voids, although uncertainty will rarely be fully removed. Lack of funding, for example, limits the ability to monitor populations and the effectiveness of conservation interventions. Addison *et al.* (2016, 1356), for example, write that ‘incomplete ecological understanding…and a lack of fit-for-purpose monitoring and research are hindering...evidence-based management’.

Lastly, ‘stakeholders not valued, considered, or opposed by interventions’ (B8) was listed in the top ten barriers. Existing research suggests that a strong connection between scientists, policy-makers, and practitioners from project conception through to implementation is the best way of building trust and producing evidence (Shanley and Lopez, 2009). Conservation is much more than a technical issue, affecting millions of people’s livelihoods around the world. Ensuring that local stakeholders are included and valued in decision-making is therefore important, and conservation scientists have suggested co-production (Beier *et al.*, 2017), or at least co-assessment (Sutherland *et al.*, *in press*), as good ways of doing conservation (Addison *et al.*, 2016; Duchelle *et al.*, 2009).

**References cited (only those not cited in main text):**

Addison, P. F. E., Cook, C. N., and de Bie, K., 2016. Conservation practitioners’ perspectives on decision triggers for evidence-based management, *Journal of Applied Ecology* **53**: 1351-1357

Bayliss, H.R., Wilcox, A., Stewart, G.B. & Randall, N.P. (2011). Does research information meet the needs of stakeholders? Exploring evidence selection in the global invasive species community, *Evidence and Policy*, 8, 37–56

Beier, P., Hansen, L. J., Helbrecht, L., and Behar, D. 2017. A How-to Guide for Coproduction of Actionable Science, *Conservation Letters* **10** (3): 288-296

Duchelle, A. E., Biedenweg, K., Lucas, C., Virapongse, A, Radachowsky, J. *et al.,* 2009. Graduate Students and Knowledge Exchange with Local Stakeholders: Possibilities and Preparation, *Biotropica* **41** (5): 578-585

McNie, E. C. 2007. Reconciling the supply of scientific information with user demands: an analysis of the problem and review of the literature, *Environmental Science & Policy* **10** (1): 17-38

Milner-Guland, E. J., Fisher, M., Browne, S., Redford, K. H., Spencer, M., and Sutherland, W. J. 2009. Do we need to develop a more relevant conservation literature?, *Oryx* **44** (1): 1-2

Shanley, P. and López, C. 2009. Out of the Loop: Why Research Rarely Reaches Policy Makers and the Public and What Can be Done, **41** (5): 535-544

**S6 Supporting quotes**

Q1 ‘Because the interest in conservation in Chile has not been massive, there are very few politicians who are attempting to generate such space and resources, and there is a very strong opposition.’ (Policy position, Chile)

Q2 ‘Policy is driven by public interest and the public does not care enough about the environment to make it a political priority.’ (Research scientist, Australia)

Q3 ‘Compiling more scientific facts there does not help, as long as the politicians are not really trying to change anything. My feeling is that public education is very important, and the basis of everything.’ (Policy position, Germany)

Q4 “the survey is naïve in believing that the Brazilian political sphere would change behaviour towards conservation after better access to scientific knowledge’. (Research scientist, Brazil)

Q5 ‘We need a revolution throughout society.” (activist working in Africa and South America)

Q6 ‘Bring about revolution (frankly, given the problem that exists, apart from a catastrophe I cannot really see what is going to change the system. It is not only a problem relating to science).’ (Research scientist, France)

Q7 ‘Creating awareness in the common masses as the attitude that is bred and established in the society forms the basis of thoughts, understanding and activities of any individual.’ (Practitioner, India)

Q8 ‘conservation will only become a political priority when the people demand it to become so’. (Research scientist, Australia)

**Supplementary figures**

**Figure S1**


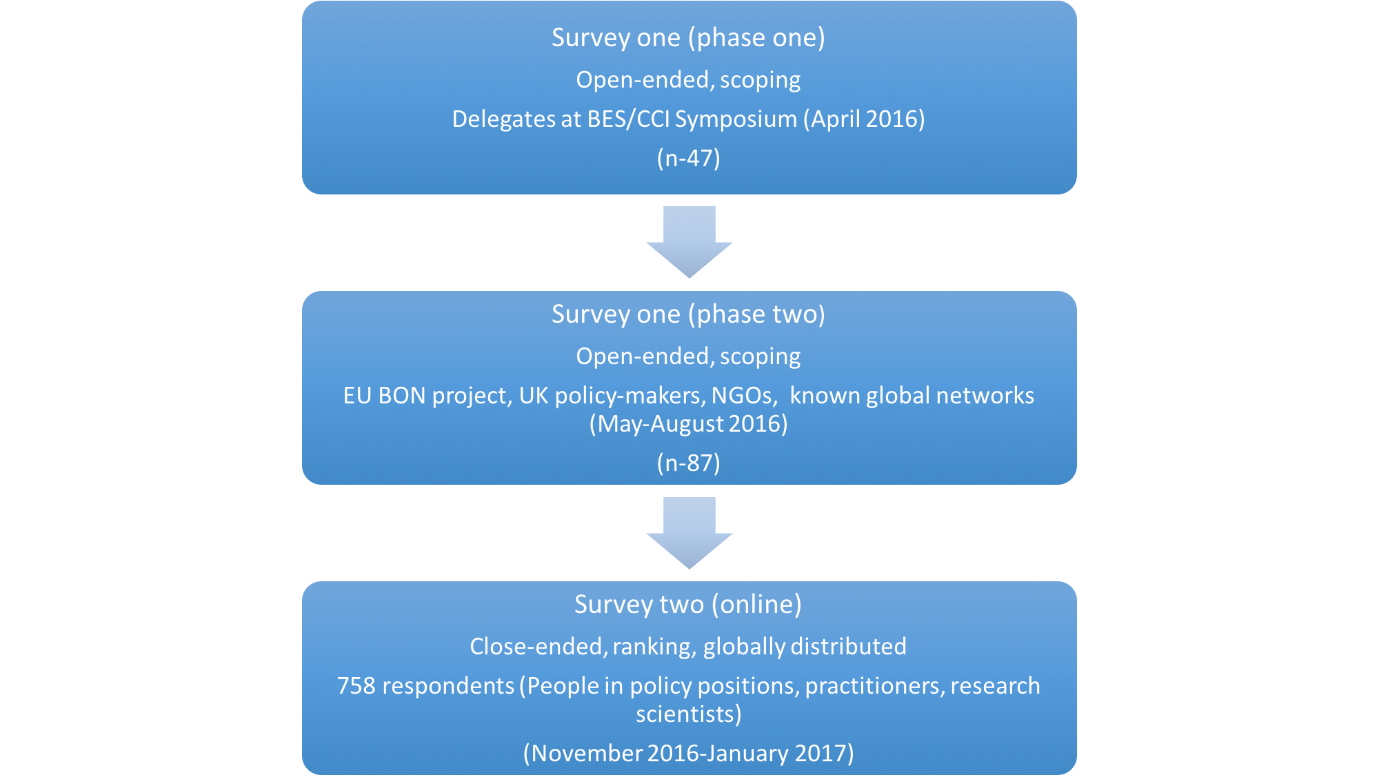


Figure S1: Flow diagram illustrating the survey methodology

**Figure S2**

**
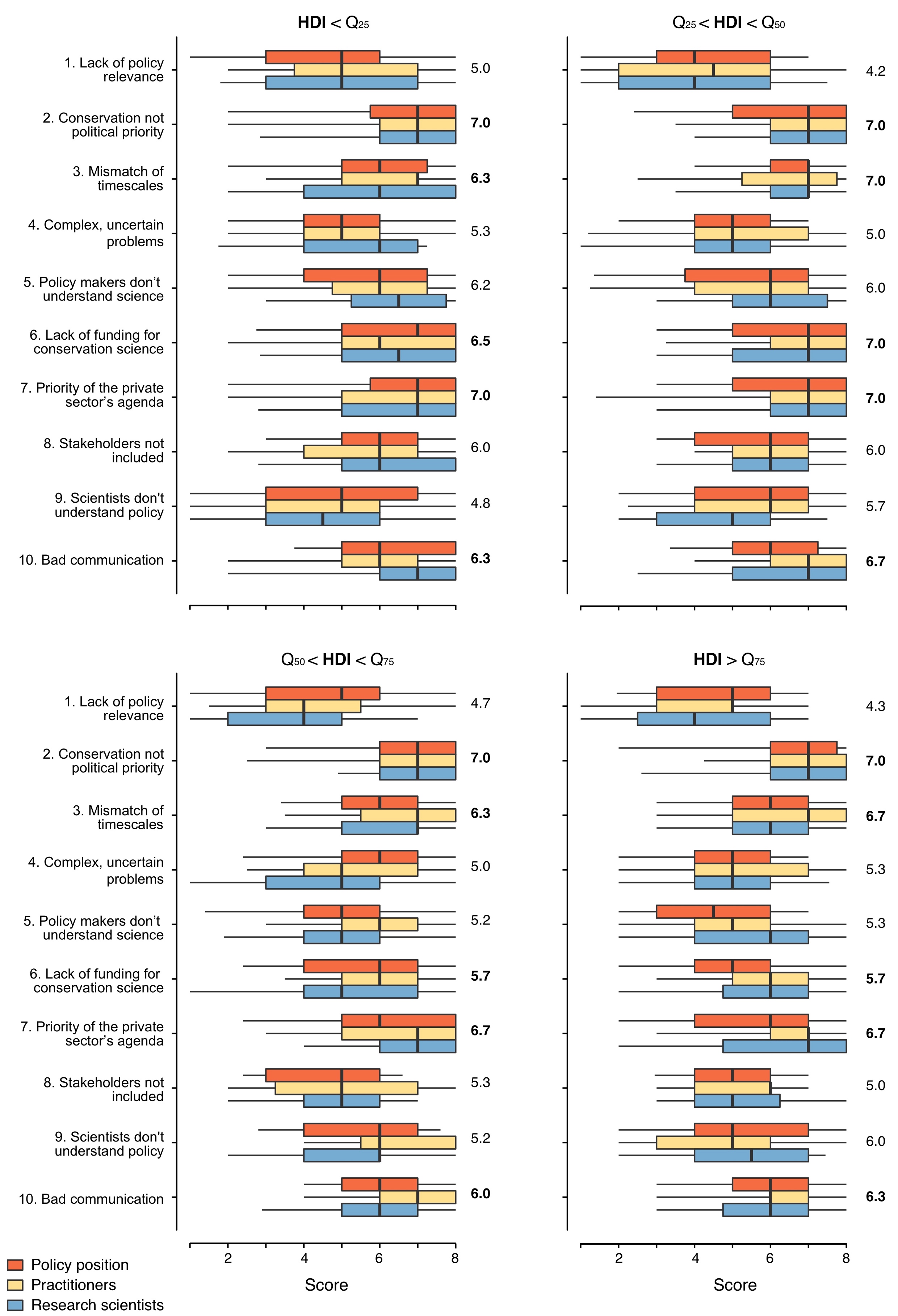
**

Figure S2: Ranking of barriers by role according to Human Development Index

**Figure S3**

Figure S3: Proportion of different roles (Red: Policy position, Yellow: practitioners, Blue: Policy position) experiencing the barriers

**Figure S4**

Figure S4: Proportion of male and female respondents to the online survey by role

**Figure S5**


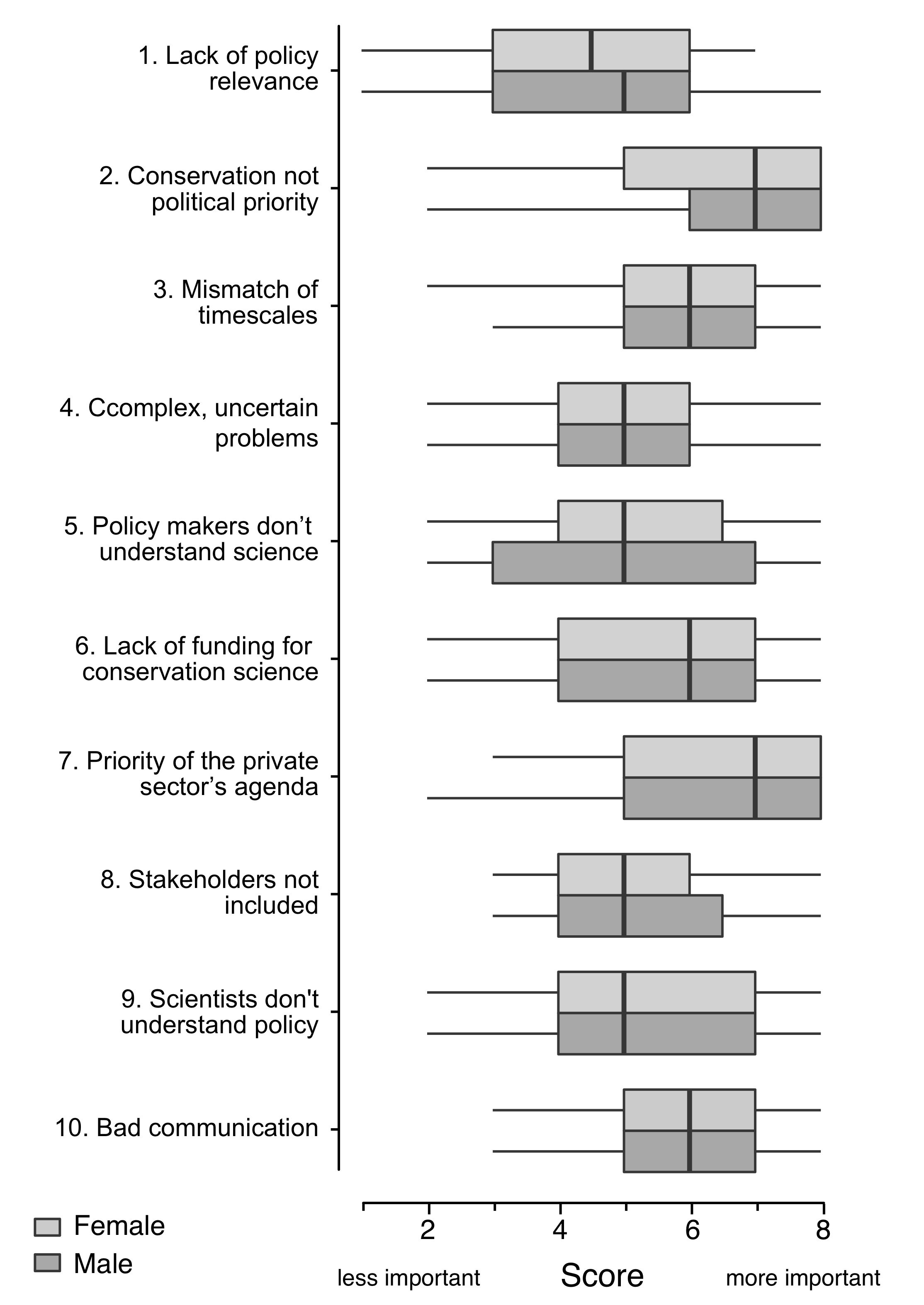


Figure S5: Ranking of barriers by gender

**Figure S6**

Figure S6: Years of experience of online survey respondents by role

**Supplementary tables**

**Table S1**

| Stage 1 (merge roles from online survey) |  |  | Stage 2 (responses from 'other' merged) | Red - excluded from analysis |
| --- | --- | --- | --- | --- |
| **Role** | **Role simplified** |  | **Role** | **Role simplified** |
| Academics (including postdocs and research associates) | Research scientist |  | Knowledge Broker | Knowledge Broker |
| Student | Student |  | Activist | Practitioner |
| Scientist (in statutory agency/government) | Policy position |  | Consultant/Environmental consultant | Practitioner |
| Practitioner (government or statutory agency) | Practitioner |  | Project Manager | Practitioner |
| Practitioner (NGO) | Practitioner |  | Ornithologist | Practitioner |
| Policy-maker (NGO) | Policy position |  | Tourism professional | Practitioner |
| Policy-maker (government) | Policy position |  | Engineer | Practitioner |
|  |  |  | Volunteer | Practitioner |
|  |  |  | Private Sector | Practitioner |
|  |  |  | Professional | Practitioner |
|  |  |  | Data Manager | Practitioner |
|  |  |  | Tool Developer | Practitioner |
|  |  |  | Commentator | Practitioner |
|  |  |  | Photographer | Practitioner |
|  |  |  | Educator | Practitioner |
|  |  |  | Scientist in NGO | Research Scientist |
|  |  |  | Manager of science | Research Scientist |

Table S1: Process followed for categorising roles

**Table S2**

| **Phase 1 - iteration 1 respondents – BES/CCI symposium (n-47)**  (total no. of delegates, 250, therefore response rate 19%) |
| --- |
| **Role** |
| Academics/research scientists (17) |
| Policy position (3) |
| Practitioners (9) |
| Student (17) |
| Knowledge broker (1) |
|  |
| **Phase 1 - iteration 2 respondents various networks (n-87)**  (not possible to determine a total population nor a response rate) |
| **Role** |
| Academics/research scientists (36) |
| Policy position (30) |
| Practitioner (12) |
| Student (7) |
| Publisher (2) |
|  |
| **Years of experience (mean)** |
| 13 |
|  |
| **Years of experience (median)** |
| 11.5 |
|  |
| **Individual countries covered** |
| Australia, Belgium, Botswana, Bulgaria, Canada, Chile, China, Ecuador, Finland, France, Germany, Italy, Japan, Kenya, Malaysia, Mozambique, Namibia, Nigeria, Poland, Portugal, Spain, Sri Lanka, Sweden, Switzerland, Syria, The Netherlands, Uganda, UK, USA, Zimbabwe |

Table S2: List of phase one survey respondents by role, years of experience, and country of work

**Table S3**

| **Barriers** | **Count** |
| --- | --- |
| Bad communication between scientists and policy-makers | 75 |
| Lack of policy relevant science | 46 |
| Conservation not a political priority | 44 |
| Mismatch of timescales | 24 |
| Complex, uncertain problems | 22 |
| Policy-makers don't understand science | 19 |
| Lack of funding for science | 15 |
| Priority of the private sector’s agenda over conservation | 14 |
| Stakeholders not valued, considered, or opposed by interventions | 14 |
| Scientists don't understand how policy is made | 7 |
|  |  |
| **Solutions** |  |
| More collaboration between scientists and policy-makers (e.g. meetings, seminars, projects) | 36 |
| Better incentives for academics to focus on policy/practice relevant research | 33 |
| Tailor evidence to audience - e.g. blogs, summaries, simple language, open access | 28 |
| Demonstrate benefits of conservation (inc. economic value) | 26 |
| Improve policy education of young scientists/scientists | 14 |
| More knowledge brokers (and system for it) | 11 |
| Trans-disciplinary science to be encouraged | 11 |
| Train policy-makers in science | 10 |
| Train scientists in communication skills | 10 |
| More funding for science | 8 |
| Better stakeholder outreach | 7 |
| More scientists working in policy positions | 7 |
| Build trusted relationships with key decision-makers | 5 |
| Scrutinise policy | 5 |
| Science outreach to public | 4 |

Table S3: Ranking of barriers and solutions from phase one survey

**Table S4**

| Bad communication between scientists and policy-makers |
| --- |
| Bad governance structures |
| Bad science |
| Bandwagon issues sideline important issues |
| Brexit |
| Complex, uncertain problems |
| Conservation not a political priority |
| Corruption |
| Difficult to change behaviour |
| Few distinct policies for aquatic and terrestrial conservation |
| Intangible impacts of change |
| Lack of expert consensus |
| Lack of funding for NGOs |
| Lack of funding for science |
| Lack of multi-disciplinary research |
| Lack of piloting of schemes to provide grounded evidence |
| Lack of policy relevant science |
| Lack of public interest |
| Lack of trust in science |
| Language |
| Media poor communication |
| Misinformed public |
| Mismatch of timescales |
| Not enough knowledge brokers |
| Policy-makers are too conservative for change |
| Policy-makers don't understand science |
| Priority of the private sector’s agenda over conservation |
| Prioritising solutions is lacking |
| Scientists don't understand how policy is made |
| Stakeholders not valued, considered, or opposed by intervention |
| Uncertainty fallacy |
| Values influence decision-making |

Table S4: All barriers mentioned in the phase one survey

**Table S5 (a) and (b)**

(a) Excluding students, **210 respondents stated they had experienced barriers**, ranking them in the following order:

1. **Bad communication between scientists and policy-makers**
2. **Not political priority**
3. **Mismatch of timescales**
4. **Priority of the private sector’s agenda over conservation**
5. **Lack of funding for conservation science**
6. Policy makers don't understand science
7. Scientists don't understand how policy is made
8. Complex, uncertain problems
9. Stakeholders not included
10. Lack of policy relevant science

(b)

| **Barrier rank** | **Ranked** | **Experienced (most common = 1)** |
| --- | --- | --- |
| Conservation not a political priority | 1 | 2 |
| Priority of private sector’s agenda | 2 | 4 |
| Mismatch of timescales | 3 | 3 |
| Lack of funding for conservation science | 4 | 5 |
| Bad communication | 5 | 1 |

Table S5: (a - top) Ranking of most experienced barriers, and (b - bottom) Overall barrier rank versus ranking of how highly experienced it was

1. This total figure included 53 academics/research scientists, 33 people in policy positions, and 21 practitioners. 24 students also responded, but responses from this group were lower for the second online survey, and thus their responses are not included in the final analyses [see Table S2]. [↑](#footnote-ref-1)
