# Supplementary material for: The major barriers to evidence‐informed conservation policy and possible solutions
Source: Conserv Lett. 2018 May 8;11(5):e12564. doi: 10.1111/conl.12564 (PMC6473637; doi:10.1111/conl.12564)
Supplement: Supplementary file 2 — Translated Abstract [file CONL-11-na-s002.zip › Arabic.pdf]

Arabic

كثيراً ما تعاني عمليات اتخاذ القرار بشأن سياسات الحفظ بشكل عام من فجوات في الأدلة والمعطيات، مما يعيق اتخاذ القرارات الفعالة. وفي مجال حفظ الطبيعة بشكل خاص، سعت دراسات عدة إلى التعرف على أسباب عدم استناد السياسات، في حالات كثيرة، إلى الأدلة، إلا أن تلك الدراسات ركزت على الديمقراطيات الغربية، واعتمدت على عيّنات بحثية صغيرة نسبياً. ولهذا، وبغرض الارتقاء بفهمنا لأوجه التفاوت والتحديات العالمية في هذا المجال، أعددنا مسحاً عالمياً يهدف إلى تحديد أهم معوقات الاستفادة من علوم الحفظ عند وضع السياسات المعنية، وما هي حلولها، حيث جمّعنا عبر المسح آراء 758 شخصاً من العاملين في ميادين السياسات والممارسة و البحوث، من 68 دولة وبست لغات. ونوضح في دراستنا أنه، وخلافاً للرأي السائد، تتفق مختلف الدوائر المعنية حول كيفية دمج علوم الحفظ في عملية وضع السياسات، و هي بوادر تحفزنا على التفاؤل. وقد أشار المشاركون في الاستبيان إلى أن العائق الرئيسي هو اعتبار قضايا الحفظ من الأولويات الدنيا، ونوهوا إلى أن الحل قد يكمن في إشهار وتعميم معطيات الحفظ، لذا وجب تركيز أولويات العمل على إقناع عامة الجمهور بأهمية قضايا الحفظ، مما سيسهم في تحفيز صانعي السياسات بتبني سياسات لحفظ البيئة طويلة الأمد.
