# Supplementary material for: The major barriers to evidence‐informed conservation policy and possible solutions
Source: Conserv Lett. 2018 May 8;11(5):e12564. doi: 10.1111/conl.12564 (PMC6473637; doi:10.1111/conl.12564)
Supplement: Supplementary file 2 — Translated Abstract [file CONL-11-na-s002.zip › Bengali.pdf]

জীব বৈচিত্র্য সংরক্ষণ নীতি এবং সিদ্ধান্ত অনেক সময় বৈজ্ঞানিক প্রমানের ভিত্তিতে নেওয়া হয়না। এর ফলে সংরক্ষণ সিদ্ধান্ত কার্যকর না হওয়া স্বাভাবিক। বৈজ্ঞানিক অনুসন্ধানের ফল কেন নীতিতে পরিণত হয়না এবং তার প্রতিকার জানার গবেষণা প্রধানত পশ্চিমা গণতান্ত্রিক দেশেই হয়ে এসেছে, অপেক্ষাকৃত স্বল্প নমুনার ভিত্তিতে। পৃথিবীর সামগ্রিক জীব বৈচিত্র্য বোঝা, তার প্রতিবন্ধকতা এবং প্রতিকারকে নীতি আয়োধের অন্তর্গত করার জন্য এই নতুন গবেষণারয় প্রয়াস। আমরা একটা বিশ্বব্যাপী সমীক্ষার মাধ্যমে ৭৫৮ মানুষের মতামত গ্রহণ করি। ছয়টি ভাষায় এই সমীক্ষার অনুবাদ করা হয় এবং পৃথিবীর ৬৮টি দেশ থেকে আমরা মতামত সংগ্রহ করি (গবেষণাকারী, পরিকল্পক এবং নীতি নির্ধারকদের থেকে)। এই গবেষণার মাধ্যমে আমরা দেখাতে পারি যে বিভিন্ন দেশ, ভাষা, জাতি, বয়েস এবং পেশার মানুষে রা এক্ষেত্রে অনেকটাই সমমনস্ক। শাসন প্রণালীতে জীব বৈচিত্র্য সংরক্ষণ পূর্বাধিকার না পাওয়া একটি বড় প্রতিবন্ধক হিসেবে ধরা পরে। উপসংহারে বলা যেতে পারে যে জীব বৈচিত্র্য বৈচিত্র সংরক্ষণ মূলধারায় নিয়ে আসাটা এর সবচেয়ে বড়ো সমাধান। সুতরাং জনসাধারণকে জীব বৈচিত্র্যের উপকারিতা এবং তার অবর্তমানে আমাদের কি ক্ষতি হতে পারে তা জানানো একান্ত প্রয়োজন। পরবর্তীকালে তা নীতি নির্ধারকদের দীর্ঘ মেয়াদী পরিবেশগত নীতির পক্ষে সিদ্ধান্তও নিতে সাহায্য করতে পারে।
