# Supplementary material for: The major barriers to evidence‐informed conservation policy and possible solutions
Source: Conserv Lett. 2018 May 8;11(5):e12564. doi: 10.1111/conl.12564 (PMC6473637; doi:10.1111/conl.12564)
Supplement: Supplementary file 2 — Translated Abstract [file CONL-11-na-s002.zip › Punjabi.pdf]

ਬਚਾਅ ਦੀ ਨੀਤੀ ਦੇ ਫੈਸਲੇ ਸਬੂਤ ਦੀ ਘਾਟ ਭੁਗਤ ਸਕਦੇ ਹਨ, ਜਿਸ ਨਾਲ ਅਸਰਦਾਇਕ ਫੈਸਲਾ ਲੈਣ ਤੇ ਅਸਰ ਪੈ ਸਕਦਾ ਹੈ। ਕੁਦਰਤੀ ਬਚਾਅ ਦੇ ਸੰਬੰਧ ਵਿਚ, ਜੋ ਅਧਿਐਨ ਇਸ ਗੱਲ ਦੀ ਜਾਂਚ ਕਰ ਰਹੇ ਹਨ ਕਿ ਕਿਉਂ ਨੀਤੀ ਆਮਤੌਰ ਤੇ ਸਬੂਤ ਦੀ ਜਾਣਕਾਰੀ ਨਹੀਂ ਦਿੰਦੀ ਹੈ ਉਹ ਪੱਛਮੀ ਲੋਕਤੰਤਰ ਤੇ ਧਿਆਨ ਕੇਂਦ੍ਰਿਤ ਕਰਦੇ ਹਨ, ਜਿਥੇ ਮੁਕਾਬਲਤਨ ਛੋਟੇ ਨਮੂਨੇ ਪੇਸ਼ ਕੀਤੇ ਜਾਂਦੇ ਹਨ। ਬਿਹਤਰ ਢੰਗ ਦੇ ਨਾਲ ਗਲੋਬਲ ਫਰਕ ਅਤੇ ਚੁਨੌਤੀਆਂ ਨੂੰ ਸਮਝਣ ਲਈ, ਨੀਤੀ ਵਿਚ ਬਚਾਅ ਦੇ ਵਿਗਿਆਨ ਦੀ ਵਰਤੋਂ ਕਰਨ ਦੇ ਉਦੇਸ਼ ਨਾਲ ਵੱਧ ਰੁਕਾਵਟਾਂ ਅਤੇ ਸੁਲਝਾਉਣ ਦੇ ਤਰੀਕਿਆਂ ਦੀ ਪਛਾਣ ਕਰਨ ਦੇ ਲਕਸ਼ ਨਾਲ ਅਸੀਂ ਇਕ ਗਲੋਬਲ ਸਰਵੇਖਣ ਨੂੰ ਸਥਾਪਿਤ ਕੀਤਾ ਹੈ। ਇਸ ਨੇ ਨੀਤੀ, ਅਭਿਆਸ ਅਤੇ ਖੋਜ ਵਿਚ 68 ਦੇਸ਼ਾਂ ਤੋਂ 758 ਲੋਕਾਂ ਦੇ ਛੇ ਬੋਲੀਆਂ ਵਿਚ ਵਿਚਾਰ ਲਿੱਤੇ ਹਨ। ਇਥੇ ਅਸੀਂ ਇਹ ਗੱਲ ਵਿਖਾਉਂਦੇ ਹਾਂ, ਕਿ ਆਮ ਮਾਨਤਾ ਦੇ ਉਲਟ, ਸਮੂਹਾਂ ਵਿਚ ਇਹ ਸਹਿਮਤੀ ਹੈ ਕਿ ਨੀਤੀ ਵਿਚ ਬਚਾਅ ਵਿਗਿਆਨ ਨੂੰ ਕਿਵੇਂ ਸ਼ਾਮਿਲ ਕੀਤਾ ਜਾਵੇ, ਅਤੇ ਇਸ ਕਰ ਕੇ ਆਸ਼ਾਵਾਦੀ ਗੱਲ ਲਈ ਗੁੰਜਾਇਸ਼ ਹੈ। ਬਚਾਅ ਲਈ ਘੱਟ ਪ੍ਰਾਥਮਿਕਤਾ ਦੇ ਸੰਬੰਧ ਵਿਚ ਜੋ ਰੁਕਾਵਟਾਂ ਹਨ ਉਨ੍ਹਾਂ ਨੂੰ ਮਹੱਤਵਪੂਰਣ ਸਮਝਿਆ ਗਿਆ, ਜਦੋਂ ਕਿ ਬਚਾਅ ਨੂੰ ਮੁੱਖ ਧਾਰਾ ਵਿਚ ਜੋੜਨ ਨੂੰ ਇਕ ਮੁੱਖ ਸੁਲਝਾਉ ਦੇ ਤੌਰ ਤੇ ਪ੍ਰਸਤਾਵਿਤ ਕੀਤਾ ਗਿਆ। ਇਸ ਲਈ, ਬਚਾਅ ਦੀ ਮਹੱਤਤਾ ਨੂੰ ਲੋਕਾਂ ਨੂੰ ਸਮਝਾਉਣ ਦੇ ਵਿਸ਼ੇ ਦੇ ਤੌਰ ਤੇ ਉਸ ਵਲ੍ਹ ਧਿਆਨ ਦੇਣਾ ਚਾਹੀਦਾ ਹੈ, ਜਿਸ ਨਾਲ ਨੀਤੀ ਬਣਾਉਨ ਵਾਲਿਆਂ ਤੇ, ਵਾਤਾਵਰਣਕ ਦੇ ਸਮਰਥਨ ਵਿਚ ਲੰਮੇਂ ਸਮੇਂ ਦੀਆਂ ਨੀਤੀਆਂ ਨੂੰ ਅਪਣਉਨ ਲਈ ਪ੍ਰਭਾਵ ਪਵੇਗਾ।
