# Supplementary material for: The major barriers to evidence‐informed conservation policy and possible solutions
Source: Conserv Lett. 2018 May 8;11(5):e12564. doi: 10.1111/conl.12564 (PMC6473637; doi:10.1111/conl.12564)
Supplement: Supplementary file 2 — Translated Abstract [file CONL-11-na-s002.zip › Translations.pdf]

Translated abstracts

Language **Page**

Portuguese…………………………………………………………………………...2

Japanese…………………………………………………………………………...3

German……………………………………………………………………………….4

Spanish……………………………………………………………………………….5

Russian……………………………………………………………………………….6

French…………………………………………………………………………...7

Hindi……………………………………………………………………………….8

Chinese (Mandarin)……………………………………………………………..9

Hungarian (Magyar)…………………………………………………………….10

Please note than Bengali, Arabic, and Punjabi are provided in separate PDF files

Credits for Bengali: Gargi Ganguly, Suddhasattwa Ganguly, Samarendra Nath Ganguly, Nibedita Mukherjee

Credits for Arabic: Dr Aban Shuaib

Credit for Punjabi: First Edition Translations

Portuguese

As decisões políticas para conservação da natureza podem sofrer com a falta de evidências científicas, dificultando assim tomadas de decisões que sejam realmente efetivas. Estudos que investigam as razões de tais políticas de conservação da natureza serem frequentemente não baseadas em evidências tendem a ser mais comuns em democracias do mundo ocidental, porém com relativamente pequeno número de observações. Para entender melhor os desafios e diferenças em todo o mundo, preparamos uma pesquisa que englobou vários países com o objetivo de identificar as principais dificuldades e soluções para o uso e alcance da ciência da conservação na esfera política. Obtivemos 758 participantes associados com política, prática e ciência da conservação em 68 países, e em seis diferentes idiomas. O estudo mostra que, ao contrário de crenças populares, há um consenso entre diferentes grupos sobre como a ciência da conservação pode ter um alcance maior em políticas para a conservação da natureza, gerando, dessa forma, espaço para otimismo. As dificuldades relacionadas com a baixa prioridade de conservação foram consideradas importantes, enquanto a sua popularização foi proposta como a solução principal. Consequentemente, as prioridades devem ser focadas em convencer a população em geral sobre a importância da conservação, influenciando, assim, os tomadores de decisões em adotarem políticas pró-ambientais que sejam de longo prazo.

Credit: Matheus Henrique Nunes and Maria Dias

Japanese

生物多様性の保全に関する政策では、科学的根拠の欠如が問題となり、効果的な意思決定が困難となることがある。科学的根拠に基づいた政策決定がなぜ行われないのかについてのこれまでの研究は、西欧諸国での限られた事例に基づいたものが多かった。そこで本研究では、地域による違いや世界での課題を理解することを目的とし、生物多様性の保全科学を政策で活用する際の障壁と解決策を明らかにするための調査を、世界規模で実施した。調査は 6 つの主要な言語で行われ、世界 68 か国から計 758 人の政策関係者、実務者、科学者が参加した。解析の結果、どのようにして保全科学を政策に組み込むべきかについて、立場の異なる人々の間でも意見の一致が見られた。この結果はこれまでの一般的な考えとは異なるもので、政策における保全科学の利用を促進するにあたって希望をもたらすものである。政策決定において生物多様性保全の優先順位が低いことに関する障壁は特に重要であると評価され、生物多様性保全の主流化がその解決策として提示された。今後は生物多様性保全の重要性について一般市民の理解を得ることで、政策決定者が環境保全を考慮した長期的な政策を実行するように影響を与えていくべきだと考えられる。

Credit: Tatsuya Amano

German

Das Fehlen von Evidenz kann politische Entscheidungen im Bereich des Naturschutzes beeinträchtigen und eine effektive Entscheidungsfindung behindern. Forschungsvorhaben, die der Frage nachgegangen sind warum politische Inhalte und Entscheidungen im Bereich des Naturschutzes oftmals nicht evidenzbasiert sind, haben die Tendenz auf westliche Demokratien zu fokussieren und ihre Ergebnisse auf relativ kleine Stichproben zu stützen. Um globale Schwankungen und Herausforderungen besser zu verstehen, haben wir eine globale Umfrage erstellt, mit dem Ziel, die größten Hindernisse und die besten Lösungen zur Verwendung wissenschaftlichen Wissens in der Naturschutzpolitik zu identifizieren. Die Studie umfasst die Ansichten von 758 Personen aus den Tätigkeitsbereichen Politik, Praxis und Forschung, 68 Länder und sechs Sprachen. Unsere Ergebnisse zeigen, dass - entgegen der landläufigen Meinung - zwischen den Gruppen Einigung darüber herrscht, wie wissenschaftliche Erkenntnisse in die Naturschutzpolitik eingegliedert werden sollten und daher sehr wohl Raum für Optimismus besteht. Als größtes Hindernis wird die geringe Priorität wahrgenommen, die Naturschutz beigemessen wird, wobei als beste Lösung das Mainstreaming von Naturschutz gesehen wird. Daher sollte das Augenmerk auf die Überzeugung der Öffentlichkeit von der Bedeutung des Naturschutzes gerichtet werden, wodurch politische Entscheidungsträgerinnen und Entscheidungsträger stärker beeinflusst werden umweltfreundliche langzeitmaßnahmen zu verabschieden.

Credit: Alice Vadrot

Spanish

Las decisiones políticas en materia de conservación pueden verse afectadas por la falta de evidencia, obstaculizando la efectividad de la toma de decisiones. En conservación de la biodiversidad, los estudios que investigan por qué las decisiones políticas frecuentemente carecen de evidencia científica se han centrado en democracias occidentales, con muestras relativamente pequeñas. Para comprender mejor la variación global y los retos en este ámbito, establecimos una encuesta global dirigida a identificar las principales barreras y soluciones para el uso de la ciencia de la conservación en la toma de decisiones. Recogimos los puntos de vista de 758 personas, en 68 países y en seis idiomas distintos, dedicadas a la conservación como políticos, profesionales e investigadores. Mostramos que existe un amplio consenso entre grupos sobre cómo incorporar la ciencia de la conservación en la toma de decisiones y, por consiguiente, hay lugar para el optimismo. Las barreras relacionadas con la escasa prioridad de la conservación fueron consideradas importantes, mientras que la integración política de la conservación se propuso como una solución clave. Por lo tanto, la prioridad está en convencer al público de la importancia de la conservación como problema, lo que influirá entonces a los gobernantes a adoptar políticas pro-ambientales a largo plazo.

Credit: Juan P. González-Varo and América Paz Durán

Russian

Решения в области политики охраны природы и рационального природопользования могут страдать от недостатка доказательств, что затрудняет эффективное принятие решений. В сфере охраны природы исследования проблем нехватки использования данных научных исследований при формировании политики были сосредоточены в основном на демократических странах Запада, при относительно малой выборке. Чтобы лучше понять глобальные перемены и трудности, мы организовали глобальное исследование, призванное установить самые сильные барьеры и найти решения для использования в политике научных данных об охране природы и рациональном природопользовании. Мы собрали мнения 758 человек из 68 стран, говорящих на шести языках и занимающихся политикой, практикой и научной деятельностью. В исследовании мы демонстрируем, что, вопреки расхожему мнению, разные группы сходятся в том, как следует использовать в политике научные данные об охране природы и рациональном природопользовании, и это дает повод для оптимизма. Важными были признаны барьеры, связанные с низким приоритетом охраны природы, а ключевым решением названо выдвижение охраны природы на первый план. Таким образом, приоритет следует отдать убеждению общественности в важности охраны природы, что заставит политиков принять долгосрочные стратегии, учитывающие задачи охраны окружающей среды.

Credit: First Edition Translations (translated) and Sarah Ivory (checked)

French

Certaines décisions politiques environnementales peuvent s'avérer inefficaces faute de preuves. En matière de conservation de la nature, les études sur les raisons de ce manque de données probantes ont eu tendance à se concentrer sur des démocraties occidentales, avec des informations disponibles relativement restreintes. Afin de mieux comprendre les variations et les défis globaux, nous avons mené une étude mondiale visant à identifier les barrières supérieures et les solutions à l'utilisation de la science de conservation dans la politique. Nous avons recueilli les avis de 758 personnes actives dans les domaines de la politique, la pratique et la recherche dans 68 pays et six langues. Nous démontrons ici qu'à l'encontre de toute idée reçue, les opinions convergent quant au mode d'intégration de la science de conservation dans la politique et qu'il y a donc lieu d'être optimiste. Alors que les barrières à l'origine de la faible priorité accordée à la conservation étaient perçues comme des obstacles majeurs, la sensibilisation à la conservation a été proposée comme solution-clé. Il s'agit donc de convaincre le public de l'importance de la conservation afin d'inciter les décideurs politiques à adopter des politiques à long terme en faveur de l'environnement.

Credit: First Edition Translations (translated) and Sarah Ivory (checked)

Hindi

संरक्षण नीति संबंधी निर्णयों में साक्ष्य की कमी, निर्णयप्रणाली को प्रभावी बनने से वंचित रख सकती है। प्रकृति संरक्षण में नीति का अकसर साक्ष्य-सूचनापरक न होना, इसकी जाँच करने वाले अध्ययनों में पश्चिमी लोकतंत्रों पर ध्यान केंद्रित करने की प्रवृत्ति रही है। वैश्विक विभिन्नता और चुनौतियों को बेहतर ढंग से समझने के लिए, हमने एक वैश्विक सर्वेक्षण तैयार किया। नीति में संरक्षण विज्ञान के उपयोग से संबंधित प्रमुख बाधाओं और समाधानों की पहचान इस सर्वेक्षण में की गई। नीति, अभ्यास व अनुसंधान से जुड़े विचार ६८ देशों के ७५८ लोगों से छह भाषाओं में प्राप्त किये गए। इसमें हमने यह दर्शाया है कि, किस प्रकार संरक्षण नीति में विज्ञान को शामिल किया जाना चाहिए, इस बारे में उत्तरदाता-समूह में सहमति है। यह खोज प्रचलित धारणा के विपरीत है, और आशावादी बने रहने की अनुमति देता है। सर्वेक्षण में हमने यह पाया कि बाधाओं में महत्वपूर्ण है संरक्षण को कम प्राथमिकता दिया जाना, व समाधान प्रस्तावों में प्रमुख है संरक्षण को मुख्यधारा में लाना। इसलिए, जन-साधारण को संरक्षण का महत्व समझाने पर ध्यान केंद्रित किया जाना चाहिए। इससे आगे चलकर नीति निर्माताओं पर पर्यावरण-अनुकूल दीर्घकालिक नीतियाँ अपनाने का दबाव पड़ेगा।

Credit: Pinaki Bhattacharya, Nibedita Mukherjee, Ratul Dasgupta

Chinese (Mandarin)

缺乏依据可能是我们有效制定环境保护政策的一大阻碍。针对自然环境保护，在为何有关政策时常依据不足的问题上，现有研究更倾向于关注西方民主国家，且所涉及的样本量相对较小。为了更好地理解全球差异与挑战，我们发起了一项全球调查，旨在明确政策制定过程中，运用环保理论所遇到的主要困难及其解决方案。此项调查共收到 758 名来自政策制定、实施及研究领域人士的观点，他们分别来自 68 个国家或地区，使用六种不同的语言。与普遍观点相反，此次调查的结果相对乐观，对于如何将保护科学纳入政策，这些调查对象的观点不谋而合。他们普遍认为阻碍环境保护的次要问题十分重要，而主流化保护方案则被提议为解决问题的关键。因此，首要任务是让公众认识到环境保护的重要性，从而进一步影响决策者采取长期的环境友好政策。

Credit: Qiao Li

Hungarian (Magyar)

A természetvédelmi szakpolitikai döntések mögül gyakorta hiányzik a megalapozott bizonyíték, akadályozva ezzel a hatékony döntéshozatalt. Azok a természetvédelmi kutatások amelyek azt vizsgálják, vajon mi akadályozza a bizonyíték alapú gyakorlatok használatát, általában nyugati demokráciákra fókuszálnak, kevés mintaszámmal. Hogy jobban megértsük a globális alternatívákat és kihívásokat, egy nemzetközi kérdőívet állítottunk össze, ami azt célozta, hogy felmérjük a legfontosabb gátakat es sikereket, amelyek a természetvédelmi tudás szakpolitikai felhasználását akadályozzák. Ennek során 758 válasz érkezett 68 ország állami, kutatói és terepi szakembereitől, 6 különböző nyelven. Ezekből kiderült, hogy globális szinten hasonló válaszok érkeztek arra vonatkozóan, hogy hogyan lehetne a természetvédelmi tudást bevonni a jogalkotásba, ami optimizmusra ad okot. A legmeghatározóbb akadályok közé tartozik a természetvédelmi ágazat alacsony szintű prioritása, míg a legfontosabb megoldások között szintén a természetvédelem népszerűsítése áll. A legfontosabb teendők közé tartozik ezért az olyan közpolitikai csatornák kidolgozása oktatási kezdeményezések által, amelyek a hosszútávú természetvédelem kompatibilis szakpolitikák fontosságát ösztönzik.

Credit: Eszter Kovacs and Agnes Kaloczkai
